# Synthesis and Characterization of Orange-Peel-Modified Particleboards Glued with Tannin-Based Resins

**DOI:** 10.3390/ma19132858

**Published:** 2026-07-04

**Authors:** Nicola Rosa, Paola Cetera, Fadwa Yassamine Tsouri, Lorenzo Moro, Elena Colusso, Michela Zanetti, Lincoln Audrew Cordeiro, Gianluca Tondi

**Affiliations:** 1 Land Environment Agriculture & Forestry Department, University of Padua, Viale dell’Università 16, 35020 Legnaro, Italy; nicola.rosa.1@phd.unipd.it (N.R.); paola.cetera@unipd.it (P.C.); michela.zanetti@unipd.it (M.Z.); 2 Natural Bioresources Laboratory, Faculty of Nature and Life Sciences, Hassiba Benbouali University of Chlef, Route Nationale N°19, Ouled Fares 02180, Algeria; y.tsourifadwa@univ-chlef.dz; 3 Industrial Engineering Department, University of Padua, Via Gradenigo 6/a, 35131 Padova, Italy; lorenzo.moro@unipd.it (L.M.); elena.colusso@unipd.it (E.C.); 4 Technological Development Center, Federal University of Pelotas, Rua Gomes Carneiro 1, Pelotas 96010-610, Brazil; lincoln.cordeiro@ufpel.edu.br

**Keywords:** orange peel, natural formulation, bio-adhesive, bio-waste, circular economy, bio-composites, polyphenols

## Abstract

The increasing demand for sustainable materials is driving research into the valorization of agro-industrial waste as lignocellulosic alternatives in engineered wood product manufacturing. This study investigates the use of bitter orange (*Citrus aurantium* L.) peel as a bio-based filler in particleboards bonded with a bio-based tannin/hexamine adhesive. Panels were fabricated by partially substituting wood chips with orange peel powder at five levels (0, 2.5, 5, 10, and 15 wt%), hot-pressed at 180 °C for 8 min at a constant biomass-to-adhesive ratio of 9:1 (*w*/*w*). Firstly, the effect of temperature on orange peels and then the microscopic structure of the composite was observed through optical stereomicroscopy and SEM. Then, mechanical performance, water resistance, and thermal conductivity were also analyzed. Substitution of up to 10 wt% of wood chips with orange peel allowed for the maintenance of internal bond strength, modulus of rupture, and modulus of elasticity, while the 2.5 wt% formulation yielded a statistically significant improvement in axial thermal insulation. Conversely, when the amount of orange waste rises over 10 wt%, significant decreases in internal bond and modulus of rupture were recorded. These findings demonstrate that *C. aurantium* peels can be used as a viable filler for formaldehyde-free particleboards, offering a promising strategy for orange waste valorization in the wood panel industry.

## 1. Introduction

The increasing pressure on global forest resources, combined with the worsening environmental crisis driven by deforestation and greenhouse gas emissions, has prompted the scientific community to actively investigate alternative materials to replace wood, as well as modifications in the manufacturing processes of wood-based panels that affect their performance properties [1].

Particleboard is one of the most widely produced wood-based panels globally, particularly in housing construction and furniture manufacturing; particleboard is generally manufactured by blending lignocellulosic particles, including wood chips, shavings and sawing residues, with a thermosetting adhesive, forming a mat, and consolidating it under heat and pressure. Panel quality is strongly influenced by raw material characteristics such as particle size and geometry, moisture content, and species composition, as well as by processing parameters including press temperature, pressing time, and adhesive content, all of which directly affect density distribution, internal bond strength, modulus of rupture, and thickness swelling [1]. Given the growing global demand for these panels, the valorization of agro-industrial waste as lignocellulosic particles in composite panel fabrication represents a promising approach from both economic and environmental perspectives [2,3].

Numerous studies have demonstrated the technical feasibility of using agricultural residues as raw materials for particleboard manufacturing. Among the most investigated fillers are sycamore leaves [4], peach nutshells [5], rice starch [6], flax shives [7], sunflower stalks [8], kiwi pruning residues [9], walnuts wood residues [10], coir fibers [11], and vine pruning residues [12]. These raw materials offer several advantages, including abundance, renewability, low cost, and biodegradability. However, their mechanical, hygroscopic, and fire-resistance properties vary considerably depending on their chemical composition, particularly their cellulose, hemicellulose, lignin, and extractive contents [9,13]. Importantly, the literature does not converge on a universal threshold beyond which agro-waste filler content impairs mechanical performance; some studies report no significant deterioration in internal bond or bending strength at moderate substitution levels (up to 30–50 wt%), while others document a progressive and linear decline from the lowest tested concentrations, depending on the nature of the filler, its particle size distribution, and its compatibility with the adhesive system [14,15]. This variability underlines the need for case-specific optimization when introducing novel agro-waste fillers into particleboard formulations.

The choice of polymeric binder also plays a critical role. Urea-formaldehyde (UF) resin is widely used owing to its ease of processing and low cost, whereas phenol-formaldehyde (PF) resin provides superior mechanical properties and enhanced moisture resistance [2]. However, growing health and environmental concerns associated with formaldehyde emissions, a compound classified as a Group 1 human carcinogen by the International Agency for Research on Cancer, have driven increasing interest in bio-based alternatives, among which tannin-based adhesives have emerged as one of the most promising candidates.

Tannins are natural polyphenolic compounds abundant in bark, wood, and leaves of many tree species and are classified into two main families: hydrolysable tannins, primarily used in the leather industry, and condensed tannins, which are preferred for adhesive synthesis owing to their high reactivity [16]. Condensed tannins from mimosa bark (*Acacia mearnsii*), quebracho heartwood (*Schinopsis balansae*), and pine bark have been most extensively investigated for this purpose [17]. When crosslinked with hardeners such as hexamethylenetetramine (hexamine), furfural, or glyoxal, tannin-based resins have yielded particleboards compliant with EN 312 specifications for interior use, while exhibiting negligible formaldehyde emissions and superior thermal stability compared to conventional UF and PF resins [16,18,19]. The choice of hardener, however, remains a subject of debate: furfural, a bio-sourced compound derived from agricultural residues, is favored in some formulations for its renewable origin and lower toxicity compared to hexamine, but has been shown to yield lower crosslinking density and inferior mechanical performance relative to hexamine in quebracho tannin systems [18,20]. Hexamine, while more effective as a crosslinker, generates trace amounts of ammonia upon curing, which some authors consider a residual concern for indoor air quality [16]. The selection of the most appropriate hardener therefore involves a trade-off between mechanical performance, bio-based content, and emission profile that has not yet been definitively resolved in the literature.

In the specific field of citrus by-product valorization, orange-derived biomass has already been investigated as a raw material for wood-based composites. The authors of [13] manufactured polymeric composite particleboards using waste orange peel as a filler and a urea-formaldehyde/phenol-formaldehyde binder, whereas those of [21] investigated pectins recovered from orange peel residues as reinforcing agents for urea-formaldehyde adhesives. More recently, Ref. [22] produced single-layer particleboards from woody pruning residues of *Citrus sinensis* using either a conventional urea-formaldehyde resin or a hybrid urea-formaldehyde/modified-starch binder. These studies demonstrate the feasibility of incorporating orange-derived resources into panel products; however, they differ substantially from the present work in terms of the anatomical fraction used, botanical species, adhesive chemistry, filler incorporation level, and targeted material properties.

Citrus flavonoid profiles are strongly species-dependent [23,24] and, among orange varieties cultivated in Algeria, bitter orange peel has been reported to exhibit particularly high total phenolic contents and antioxidant capacity [25]. These species-dependent differences in extractives and cell-wall-associated compounds are relevant to particleboard manufacture because extractives can influence surface polarity, acidity, wettability, adhesive penetration, curing behavior, and interfacial bond development [26]. Differences in the composition and organization of pectic and lignocellulosic constituents may also affect moisture sorption, dimensional stability, heat transfer, and limited oxygen index (LOI) [27]. Accordingly, the performance of *Citrus aurantium* peel in tannin/hexamine-bonded particleboards cannot be reliably inferred from results obtained using *Citrus sinensis* biomass and therefore requires specific experimental evaluation.

One of the most critical parameters in particleboard fabrication is the filler-to-binder mass ratio, which directly influences bending strength, water absorption, and thickness swelling [2,13]. In general, increasing resin content improves mechanical resistance and reduces water absorption; however, beyond an optimal ratio, excess polymer may be extruded during hot pressing [13]. Therefore, determining the optimal *C. aurantium* peel concentration is essential for designing a high-performance and economically viable bio-composite.

The present work therefore aims to develop homogeneous particleboards based on *C. aurantium* peels at five substitution levels (0, 2.5, 5, 10, and 15 wt%), bonded with a formaldehyde-free quebracho tannin/hexamine adhesive, evaluating effects of orange peel concentration on internal bond strength, bending properties, thermal conductivity, and water resistance, with the threefold objective of (i) reducing wood consumption, (ii) valorizing orange agro-waste, and (iii) assessing whether orange peel incorporation can enhance composite performance.

## 2. Materials and Methods

### 2.1. Materials

Quebracho tannin powder (Fintan 737 B) from Silvateam Spa (S.Michele Mondovì, Italy) and mixed recycled core wood particles from Fantoni Spa (Osoppo, Italy) were kindly supplied by the companies, while a single species of orange (*Citrus aurantium*) was collected in January 2025 from a locality called Mouzaia within Blida, which is located in northern Algeria. NaOH ≥ 98% (CAS n°1310-73-2) and hexamethylenetetramine ≥99% (CAS n°100-97-0) for the adhesive production were purchased by Thermo Scientific Chemicals (Waltham, MA, USA). 

### 2.2. Processing and Characterization of Orange Peels

Orange fruits were washed with tap water and the peels were cut by hand in into pieces of approximately 3 cm before being air-dried at room temperature for 15 days, avoiding exposure to direct sunlight. The peels were then ground into a fine powder using a high-speed multifunction grinder; particle size distribution (Table 1) has been evaluated by sieving with different mesh sizes and these findings are also supported by stereomicroscopy imaging of the material. 

The resulting powder was kept in an opaque container at an ambient temperature of 20 °C until subsequent use.

FTIR analysis was conducted with a Nicolet SummitX FTIR spectrometer (Thermo Fisher Scientific, Waltham, MA, USA) on the *Citrus aurantium* peels after different temperature exposures (from 20 °C to 180 °C). Before each analysis, samples were conditioned for 1 h into a Ghibli laboratory thermostatic oven from Fratelli Galli (Fizzonasco, Italy) and subsequently stabilized for 15 min.

### 2.3. Synthesis of Tannin-Based Adhesive

Bio-adhesive was produced using quebracho tannin powder, which was weighed and solubilized in a water solution with a 1:1 *w*/*w* ratio and mechanically stirred for 5 min at 100 rpm. Subsequently, the pH of the solution was adjusted to 9.0 using a NaOH solution (33% *w*/*w*). A hexamethylenetetramine solution (33% *w*/*w*) was then added at a 1:11 (*w*/*w*) ratio.

### 2.4. Particleboards Preparation and Hot-Pressing

In Table 2 the composition of the particleboards acting as the subject of the present study is reported. The amounts were calculated to produce particleboards with target density of 750 kg m^−3^.

The biomass matrix was mixed with the adhesive together with biomass in a plastic bucket under a fume hood. Once the mixture was homogenized, it was introduced in a 40 × 40 cm mold to homogeneously distribute the wet particles over the surface. The prepressed panel was then pressed with a PT 50 hydraulic press from Bologna Presse (Monteveglio, Italy). All the bio-composites were produced in the same pressing conditions, i.e., 8 min at 180 °C using a pressing force of 2.45 MPa to achieve bio-composites with a target thickness of 1 cm.

### 2.5. Sample Preparation and Testing

**Figure 1:**
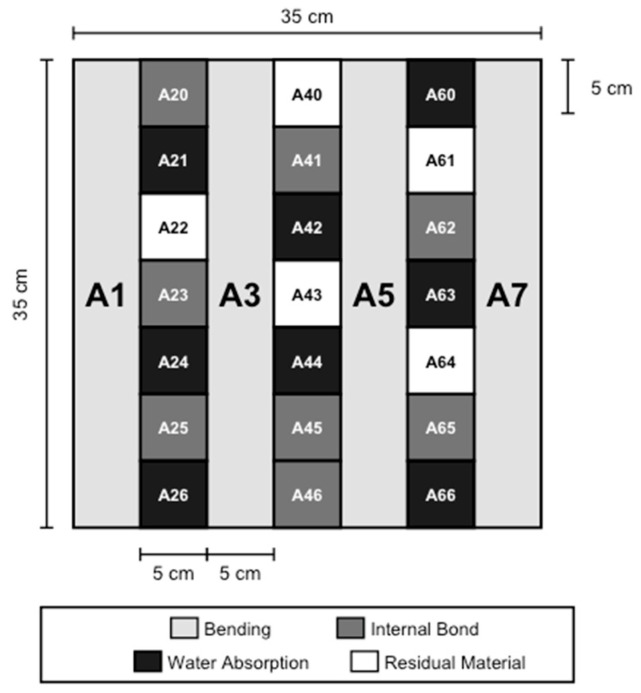
Scheme of samples production from a particleboard with an initial dimension of approximately 40 × 40 cm.

After production, panels were cooled under a laboratory hood to avoid VOC dispersion in the working environment and were subsequently trimmed and cut with a circular saw to perform mechanical testing; hence, the 5 × 5 cm and 35 × 5 cm samples were obtained with a scheme reported in Figure 1 to allow the selection of samples for all the different tests representing all the area of the produced panels.

The density of the single specimens was calculated measuring the weight and the dimensions of the stabilized sample.

The mechanical tests were performed using a Quasar 25 universal testing machine from Galdabini (Cardano al Campo, Italy). The internal bond tests were performed according to the European Norm EN 319:1993 on 5 × 5 cm samples at a crosshead speed of 2 mm min^−1^. 

The bending resistance was done according to EN 310:1993 on 5 × 30 cm samples at a crosshead speed of 2 mm min^−1^.

Water resistance was assessed by putting the 5 × 5 cm samples inside beakers filled with deionized water; a small inert weight (0.5 kg) was applied on top to prevent sample flotation and the test was carried out at laboratory room temperature. Weight and thickness of samples were measured after 1 h and 24 h of immersion time.

Axial and radial thermal conductivity were measured with a hot disk TPS 3500 (Gothenburg, Sweden) equipment using the Transient Plane Source method. Specifically, the anisotropic analysis module was applied, which requires the volumetric heat capacity, as an input parameter. In this work it was calculated by a mass-weighted average of specific heat values of the components: dry wood, water, and adhesive. Two samples of 5 × 5 cm having an average density of 766 kg m^−3^ were used for testing each composite typology. 

Microscopic imaging of the bio-composites was carried out by using a stereomicroscope Leica DMC4500; moreover, SEM (Scanning Electron Microscopy) was conducted with an ESEM (FEI Quanta 200, Eindhoven, The Netherlands) equipped with a backscattered electron detector, operating at 20 kV under low-vacuum conditions. A 1 cm^2^ representative bio-composite sample was cut and mounted on the sample holder with carbon tape. Pore size distribution has been assessed using ImageJ software (Version 1.54 g).

### 2.6. Statistical Analysis

Results are reported as mean values with Standard Deviations (St.Dev.), and 2 bio-composites were produced for each treatment to confirm consistency and reproducibility of data. For each treatment (panel typology), we tested 16 samples for the internal bond and water absorption analysis, 8 samples for the bending properties analysis, while only 3 samples for the thermal conductivity analysis due to the high accuracy (low St.Dev.) of the methodology. Outliers have been removed before statistical analysis. To assess statistically significant differences (α = 0.05) resulting from bio-composites with different orange peel concentrations, One-way ANOVA and Tukey HSD (Honestly Significant Difference) for post hoc analysis were carried out by using R software; results are reported as letters and different letters represent statistically different groups. In the case of water resistance, this approach was applied on temporal data stratification, while for temporal variation within each treatment, independent *t*-tests were performed to assess statistically significant differences; results are reported with different number of asterisks according to different number of *p*-values (“*” for *p* < 0.05, “**” for *p* < 0.01, “***” for *p* < 0.001, “ns” for *p* > 0.5). 

## 3. Results

### 3.1. FTIR

**Figure 2:**
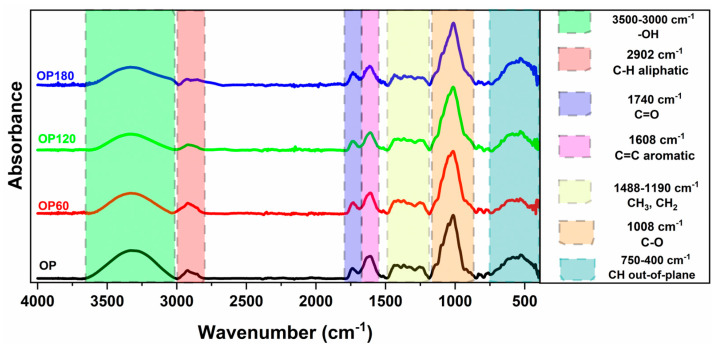
FTIR characterization of OP at different temperatures: ambient temperature at 20 °C (OP line), 60 °C (OP60 line), 120 °C (OP120 line) and 180 °C (OP180 line).

Figure 2 shows the FTIR spectra of the orange peels samples exposed to temperatures ranging from 20 to 180 °C, simulating the conditions during hot-pressing. Differences associated with thermal degradation of biomass components were observed.

A broad band between 3500–3000 cm^−1^ was attributed to O–H stretching vibrations from cellulose, pectin, hemicellulose, phenolic compounds (such as lignin and tannins), and residual water present in the sample. The band at 2902 cm^−1^ corresponds to aliphatic C–H stretching vibrations associated with lignin, cellulose, and tannins. The peak at 1740 cm^−1^ is related to carbonyl (C=O) groups mainly from pectin, hemicellulose, cellulose, and lignin [28]. The band at 1608 cm^−1^ was assigned to aromatic C=C vibrations from lignin and tannins. The region between 1488–1190 cm^−1^ is associated with aromatic and aliphatic groups, including methyl, methylene, methoxy, and aliphatic chains (CH_2_ and CH_3_) [29], 1008 cm^−1^ corresponds to C–O vibrations of alcohol and ether groups, and 750 to 400 cm^−1^ region is associated with out-of-plane vibrations of aromatic compounds (lignin, tannin) [30], as well as other components such as pectin and hemicellulose.

The thermally treated orange peel showed progressive modifications mainly in the hydroxyl and aliphatic regions, which may directly influence the development of bio-based adhesives. The flattening tendency of the O–H stretching band around 3340 cm^−1^ suggests changes in the hydrogen-bonding network due to possible partial dehydration of polysaccharides [31]. 

Simultaneously, the slight flattening observed near 2909 cm^−1^, attributed to aliphatic C–H stretching vibrations, indicates partial reorganization or degradation of polysaccharide components, contributing to modifications in the organic matrix. Such structural changes may be due to matrix cohesion [32]. In addition, moderate thermal treatment may expose reactive functional groups and promote the formation of more stable aromatic or conjugated structures [33,34].

The last observation is related to a general smoothening of the spectrum profile which suggest the loss of volatiles and the increase of macromolecules.

Overall, thermal treatment induced minor changes on the orange peels, producing a tendentially less hydrophilic matrix.

### 3.2. Bio-Composites Imaging

#### 3.2.1. Stereomicroscope

**Figure 3:**
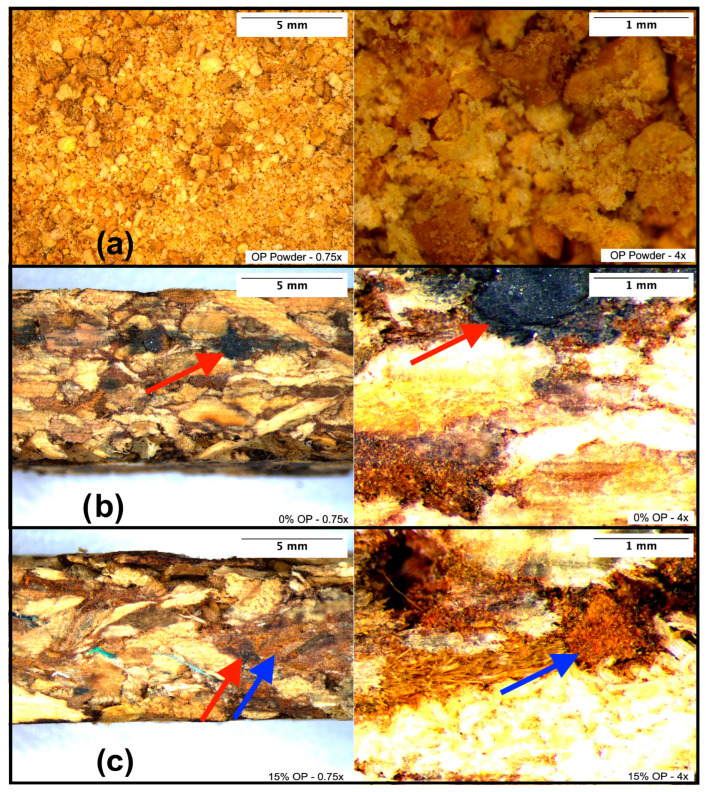
Stereomicroscope images. (**a**) OP powder with 0.75× and 4× magnification; (**b**) 0% OP sample with 0.75× and 4× magnification; (**c**) 15% OP sample with 0.75× and 4× magnification.

Cross-sectional stereomicroscope images of the bio-composite panels reveal a progressive modification of the panel microstructure with increasing *C. aurantium* peel content. Figure 3a shows the OP powder prior to incorporation into the bio-composites. In the reference panel (0% OP Figure 3b), the cross-section displays a homogeneous wood particle matrix in which the tannin adhesive is partially impregnated into the lignocellulosic substrate, as evidenced by the darkened interfacial regions surrounding individual wood chips, a feature indicative of effective adhesive penetration and consistent with the strong wood–binder interaction inferred from the mechanical results. On the other hand, the two black spots (red arrows) represent regions with higher amounts of adhesive due to a not completely homogeneous distribution along the particleboard. As OP content increases, orange peel particles become progressively identifiable within the matrix, appearing as lighter, texturally distinct regions (blue arrows) interspersed among the wood chips. At 10 and 15 wt% OP, the cross-sections reveal the formation of localized agglomerated regions, where orange peel particles cluster together rather than distributing homogeneously throughout the wood matrix. These agglomerates, characterized by irregular boundaries and reduced adhesive coverage, are consistent with the deterioration of mechanical properties observed at 15 wt% OP (Figure 3c), as particle clustering limits the effective interfacial contact area between the biomass and the tannin-based binder, thereby reducing the cohesive strength of the composite.

#### 3.2.2. SEM 

**Figure 4:**
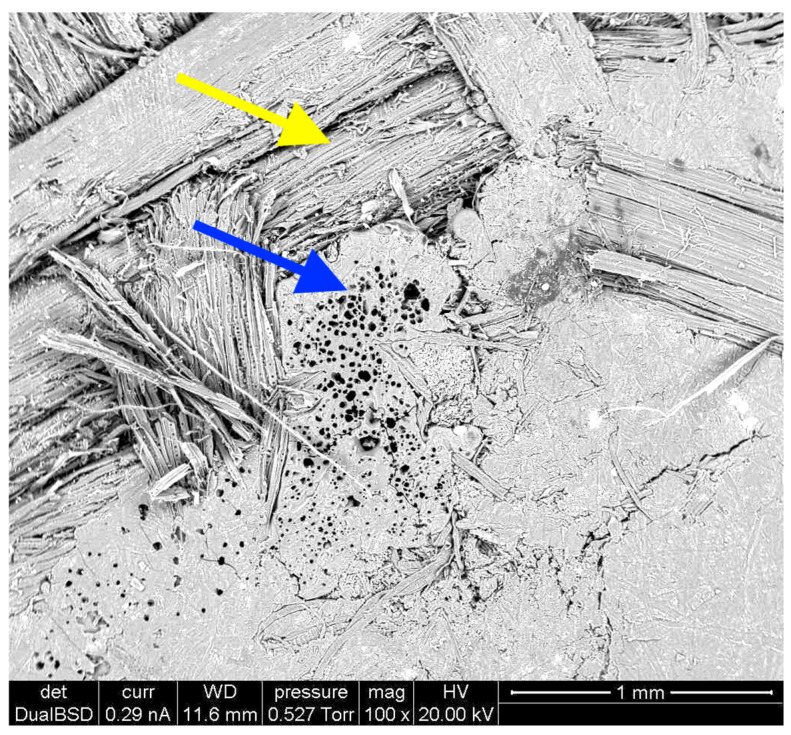
SEM image of the superficial surface of 10% OP sample. Yellow arrows indicate wood particles. Blue arrows indicate the orange peels.

The SEM analysis of the 10% OP sample in Figure 4 does not reveal any relevant visual information between wood particles (yellow arrow) and tannin-based adhesive; through this imaging technique, it is also difficult to discriminate the two materials by elemental composition, since they are both predominantly carbon-oxygen based. Conversely, at higher concentrations (10% and 15%) of OP, the bio-composite surface displays highly porous regions (blue arrow) attributable to OP agglomerates. In quantitative terms, the observable agglomerates in the 10% OP sample represent around 15% of the image’s total area; the pores in the agglomerates are around 10.7%, and they have an average size of 1.5 × 10^−4^ mm^2^ while the biggest pore detected has a size of 6 × 10^−3^ mm^2^.

### 3.3. Mechanical Properties

#### 3.3.1. Internal Bond (IB)

**Figure 5:**
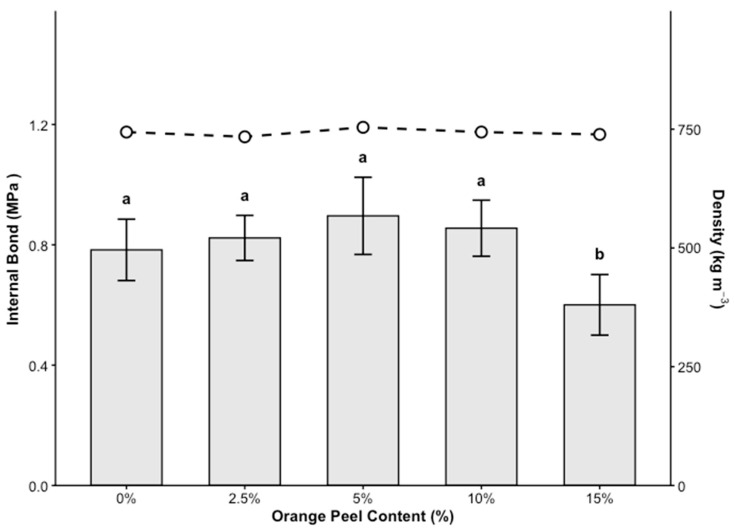
IB means (with St.Dev. bars) for particleboards with different OP content; white points represent relative densities. Tukey test between different OP particleboards: statistical groups are represented by letters.

In Figure 5, the IB results for the tested particleboards are reported. It can be observed that the internal cohesion does not modify significantly when low–medium amounts of OP are added, while a 23% reduction of IB was recorded for 15% OP. This can be attributed to the excessive orange peel content, which tends to agglomerate and does not provide the same structural behavior as wood particles. 

This is consistent with the behaviors reported for other agro-waste fillers: [35] documented a progressive deterioration of IB, MOR, and MOE in particleboards as the fraction of orange branch particles increased relative to wood, attributing this to the lower lignocellulosic content and reduced compatibility of orange-derived particles with the adhesive matrix. More broadly, [15], in a comprehensive review of seed- and fruit-based biomass boards assessed against EN 312, reported that pure agro-waste boards typically exhibit IB values below 0.62 MPa and are restricted to P1–P2 classifications, whereas hybridization with wood particles significantly enhances cohesion, which is a strategy analogous to the wood/OP blending adopted in the present study. With respect to EN 312:2010 requirements for panels in the 6–13 mm thickness range, the minimum IB for type P2 (interior use in dry conditions) is 0.40 MPa and for type P1 (general purpose, dry) is 0.35 MPa; according to their IB, the present panels can widely reach P2 classification.

Density for this mechanical test was not significantly variable, hence the bio-composites produced were sufficiently homogeneous despite a variability (St.Dev.) on the results which is common for these heterogeneous composites.

#### 3.3.2. Bending Properties

The results of the three-point bending tests are reported separately for the modulus of elasticity (MOE) and the modulus of rupture (MOR). 

**Figure 6:**
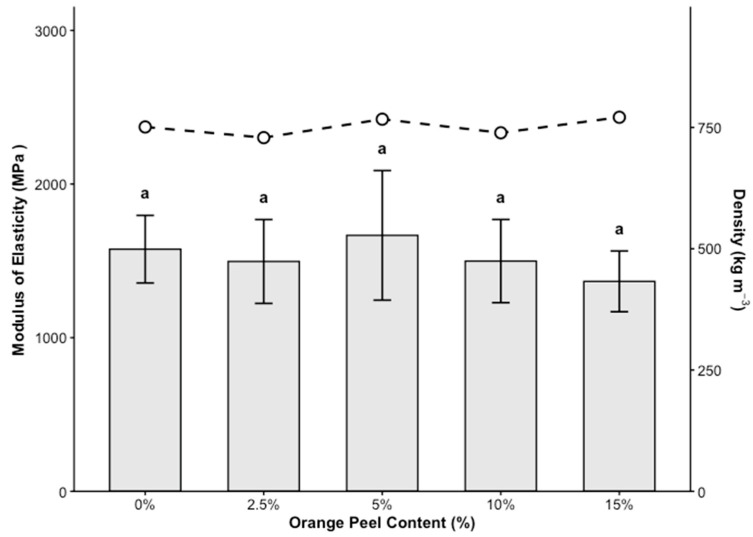
MOE means (with St.Dev. bars) for particleboards with different OP content; white points represent relative densities. Tukey test between different OP particleboards: statistical groups are represented by letters.

Figure 6 shows that MOE is statistically unaffected by OP addition, confirming that adding up to 15% OP does not alter the elastic properties (specifically the stiffness) of the bio-composite, which is primarily governed by the wood particle matrix and the tannin network. These results are consistent with the high crosslinking efficiency of hexamine as a hardener for quebracho tannin: [20] reported that quebracho/hexamine formulations achieve the highest rigidity among all tested hardeners in thermomechanical analysis (MOE = 2700 MPa), attributed to superior crosslinking density within the flavonoid network.

**Figure 7:**
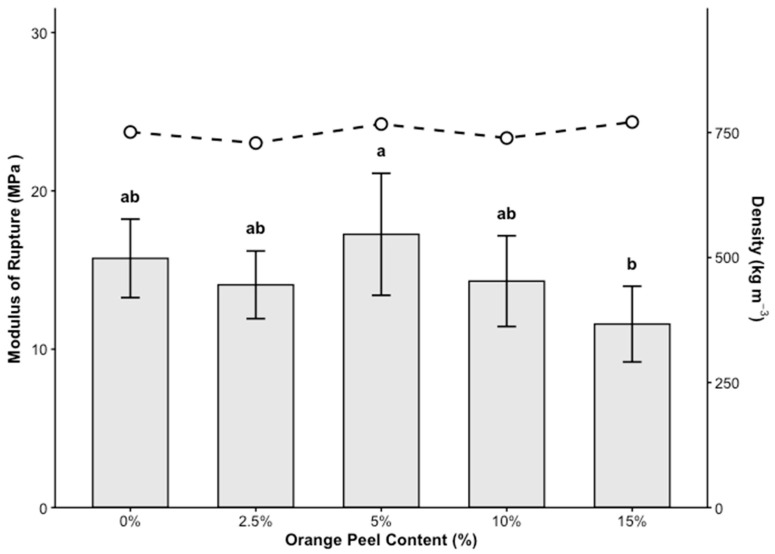
MOR means (with St.Dev. bars) for particleboards with different OP content; white points represent relative densities. Tukey test between different OP particleboards: Statistical groups are represented by letters.

MOR values (Figure 7) have no significant variation between 0% and 10% OP; therefore, low–medium concentrations of orange peel do not display any relevant effect on the flexural strength of the panels. It can be observed how the differences (not significant) between MOR mean values are in line with the oscillations (not significant) of treatment densities. The 15% OP concentration shows a 28% decrease on the performance compared to the blank (0% OP), which is significantly lower compared to the best result obtained for the 5% OP panel, and that cannot be explained by density variation; therefore, at higher concentrations (15%) orange peel negatively affects the bending properties, specifically, the maximum stress that the bio-composite can handle before rupturing, due to particle agglomeration, which is consistent with SEM observations. This trend is in agreement with findings reported by [35] for orange-branch-based particleboards and with the general pattern documented by [14], who reviewed a broad range of agricultural biomass particleboards and found that MOR and MOE systematically decrease when agro-waste particles replace wood beyond a threshold fraction, primarily due to weaker particle–adhesive interfacial bonding. With respect to tannin-bonded reference systems, [18] reported MOR = 4.9 MPa and MOE = 1417 MPa for pure quebracho/furfural particleboards, values that satisfy EN 312 P1 requirements for MOE but fall below the minimum MOR threshold for any P-type classification (11 MPa for P2, 6–13 mm thickness). The MOR values obtained in the present work for formulations containing from 0 to 10 wt% OP are thus directly comparable and potentially superior to those reported for quebracho tannin bonded with furfural, further confirming the advantage of hexamine as a more effective hardener for this tannin source.

#### 3.3.3. Water Resistance

**Figure 8:**
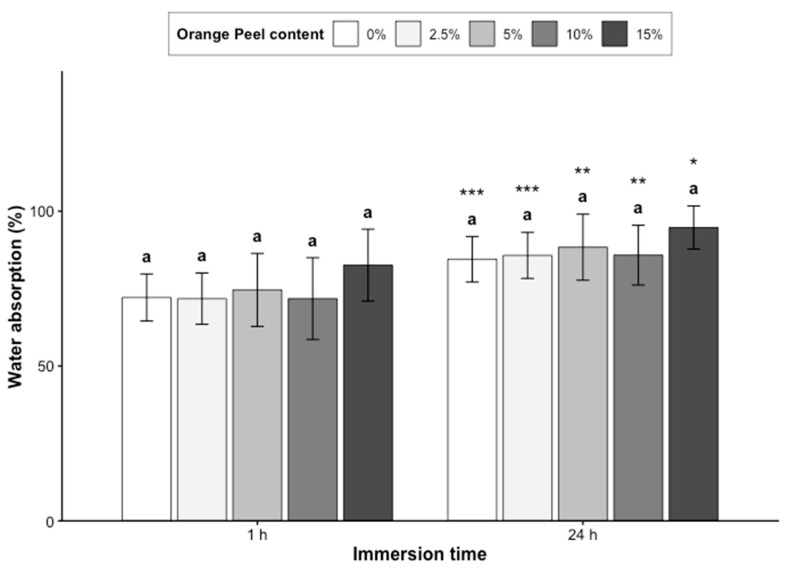
Water absorption means (with St.Dev. bars) of different particleboard typologies after 1 h and 24 h. For the 2 Tukey tests between different particleboard typologies after 1 h and after 24 h: statistical groups are represented by letters. For the 5 temporal *t*-tests on each particleboard typology: “*” for *p* < 0.05, “**” for *p* < 0.01, “***” for *p* < 0.001.

**Figure 9:**
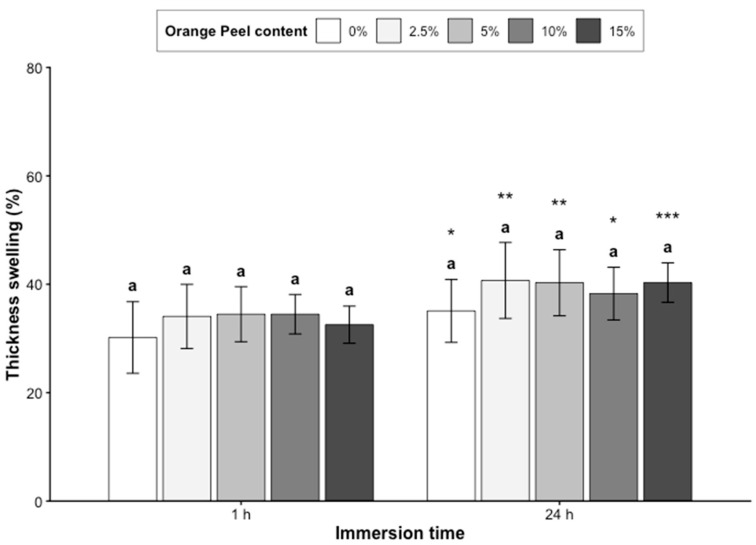
Thickness swelling means (with St.Dev. bars) of different particleboard typologies after 1 h and 24 h. For the 2 Tukey tests between different particleboard typologies after 1 h and after 24 h: statistical groups are represented by letters. For the 5 temporal *t*-tests on each particleboard typology: “*” for *p* < 0.05, “**” for *p* < 0.01, “***” for *p* < 0.001.

Water resistance is determined by water absorption (Figure 8) and thickness swelling (Figure 9) after 1 h and 24 h. Results are compared between different treatments after the same amount of time (through two independent Tukey tests) and between different immersion times within the same treatment (through five *t*-tests).

The biggest effect is detected after 1 h for each formulation of bio-composite, where most of the water is absorbed by the biomass (mean value between all the treatments is a 73% increase in weight); after 24 h, the absorbed water significantly increases compared to after 1 h in every typology of bio-composite, even though in absolute terms it is much less relevant (mean value between all the treatments is almost 87% increase in weight after 24 h). Thus, from 1 h to 24 h, the average weight increase from the beginning of the experiment is only 14%, meaning that after the first hour most of the water has already been absorbed. After the same amount of time, results do not display a significant difference between panels with different orange peel content; this implies that the introduction of this specific organic waste does not affect the kinetics of water absorption compared to the control (0% OP), although there is a certain tendency of increasing the absorption when higher amounts of OP are applied. 

Also for thickness swelling, the biggest effect is detected after 1 h for each formulation of bio-composite, where most of the water is absorbed by the biomass and the swelling occurs (mean value between all the treatments is a 33% increase in thickness); after 24 h, the thickness swelling significantly increases compared to after 1 h in every group of bio-composite, even though in absolute terms it is much less relevant (mean value across all the treatments is almost a 39% increase in thickness after 24 h). Thus, from 1 h to 24 h, the additional thickness increase with respect to the beginning of the experiment is only 6%, meaning that most of the thickness swelling occurs after the first hour.

After the same amount of time, results do not display a significant difference between panels with different orange peel content; this implies that the introduction of this specific organic waste does not affect the dimensional stability compared to the control (0% OP).

Both results for water absorption and thickness swelling are characteristic of uncoated bio-based particleboards manufactured from lignocellulosic biomass without hydrophobic surface treatments and are consistent with the hygroscopic nature of both wood particles and orange peel. 

These results, while encouraging from a comparative standpoint, reflect a known limitation of tannin-based adhesive systems in general: ref. [18] identified thickness swelling as the primary weakness of quebracho tannin particleboards, regardless of the filler type used, and attributed it to the hydrophilic nature of the polyphenolic matrix and the absence of moisture-repelling agents. Similarly, ref. [35] reported that water absorption and thickness swelling increased proportionally with orange branch content in wood–orange hybrid boards, a behavior not observed in the present study, possibly owing to the lower substitution levels tested and the finer particle size of the OP powder [13]. The use of MDI (Methylene Diphenyl Diisocianate) as a more hydrophobic binder for *Citrus sinensis* peel particleboards reported a progressive decrease in water absorption capacity from 0.35 to 0.16 g water/g material with increasing binder content, highlighting that the higher water absorption observed in the present work is primarily a consequence of the adhesive system rather than the orange peel filler. From a broader perspective, ref. [36] confirmed that agro-waste-based particleboards systematically exhibit higher water absorption and thickness swelling than conventional wood boards, owing to the greater porosity and hydrophilicity of non-woody lignocellulosic particles, a limitation that future formulations could address through surface hydrophobization of the OP filler or the addition of wax-based sizing agents. This result suggests also that during the preparation of the composite, the amount of energy applied is not enough to enhance the hydrophobicity of the orange peels as expected after FTIR study and this can be due to the fact that the wooden particles somehow protect most of the orange peels from the heat of the press.

### 3.4. Thermal Conductivity

**Figure 10:**
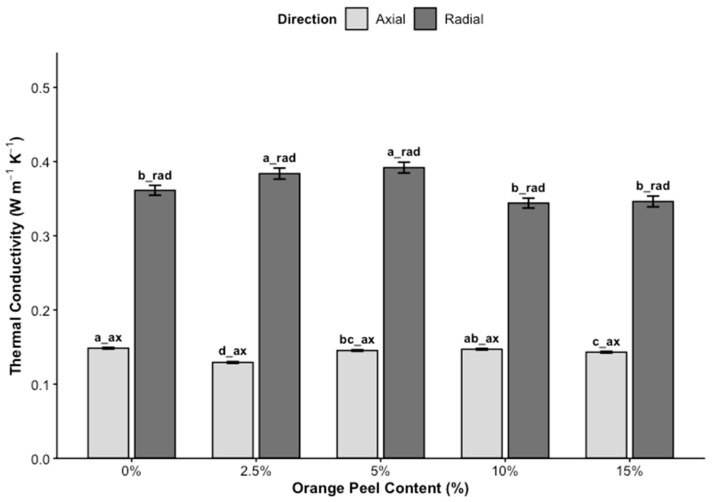
Thermal conductivity means (with St.Dev. bars) for particleboards with different OP content, both for axial and radial directions. For the 2 Tukey tests between different particleboard typologies and the same spatial direction: *statistical groups are represented by letters*.

Given the orthotropic morphology of the OP particleboard samples, thermal conductivity was evaluated in both axial and radial directions: results are shown in Figure 10. 

The orthotropic nature of the material was confirmed with axial thermal conductivity approximately one-third of the corresponding radial values.

The axial thermal conductivity values measured in the present study (ranging from 0.129 W m^−1^ K^−1^ at 2.5 wt% OP to approximately 0.148 W m^−1^ K^−1^ for the neat wood–tannin panel) are consistent with the range reported for laboratory-scale particleboards from alternative biomasses in the literature. Ref. [37] reported an axial thermal conductivity of 0.115 W m^−1^ K^−1^ for Paulownia wood particleboards, a value close to the 2.5 wt% OP optimum recorded in the present study, despite the lower density of the Paulownia boards. Ref. [38], in a study characterizing pure orange peel boards produced by thermocompression without additional adhesive, classified the resulting panels as thermal insulation materials owing to their low conductivity, attributable to the intrinsic porosity and air-trapping capacity of orange peel, a mechanism consistent with the insulation improvement observed at 2.5 wt% OP in the present work. The 13% reduction in axial thermal conductivity at 2.5 wt% OP relative to the wood–tannin reference, which proved statistically significant, can be interpreted as the result of microscopic air pockets trapped within the porous peel structure at low filler concentrations. At higher OP contents (from 5 to 15 wt%), matrix saturation and particle agglomeration progressively reduce this effect, as also inferred by SEM observations showing larger void regions at ≥10 wt% OP. For reference, particleboards produced from vine pruning waste, another agricultural lignocellulosic residue, exhibited markedly lower axial thermal conductivity values (0.064–0.068 W m^−1^ K^−1^) [39], reflecting their significantly lower density; this highlights that thermal conductivity in these systems is strongly density-dependent, and comparisons should account for panel density when evaluating insulation performance.

## 4. Conclusions

This study demonstrates the feasibility of producing particleboards bonded with a tannin-based adhesive and incorporating *Citrus aurantium* peel as a bio-based filler, suitable for interior use under dry conditions.

Thermal treatment of orange peel up to 180 °C does not induce significant chemical changes in the material; consequently, this bio-waste does not noticeably affect the binding properties of the resin during hot-press curing.

The addition of orange peels up to 10% in the particleboard does not significantly affect mechanical properties (IB, MOE, and MOR) of the bio-composite; however, when adding 15% of OP, significant reductions of mechanical properties (IB and MOR) were observed.

Thermal insulation is also similar for all formulations except for the composite having 2.5% OP: here, the thermal conductivity shows 13% decrease. 

Counter to expectation, the water resistance did not improve across different amounts of OP applied, and this was due to the low thermal modification occurring in the orange peels.

In summary, the incorporation of moderate amounts of OP promotes an improved homogenization and packing of wood chips without impairing the mechanical properties and with a simultaneous reduction in thermal conductivity. Unfortunately, this substrate does not significantly increase the water resistance. As expected, when higher proportions of OP are added (>10%), the mechanical properties decrease.

Given the large quantities of orange peel generated as agro-industrial waste, this study shows that small amounts of this bioresource can be incorporated into wood chip blends without compromising the properties of tannin-bonded particleboards. Future work will focus on extending the thermal pre-treatment of orange peel to obtain a more hydrophobic fraction, with the aim of enhancing the water resistance of the resulting composites.

## Figures and Tables

**Table 1:** Orange peel powder particle size distribution.

Particle Size	Distribution (%)
>2 mm^2^	2.6
2 mm^2^ < x < 1 mm^2^	6.1
1 mm^2^ < x < 0.5 mm^2^	22.4
0.5 mm^2^ < x < 0.3 mm^2^	11.8
<0.3 mm^2^	57.1

**Table 2:** Dry composition of the panels.

Particleboards	Wood Chips (%)	Orange Peel (%)	Adhesive (%_bm_)
0%	100		10
2.5%	97.5	2.5	10
5%	95	5	10
10%	90	10	10
15%	85	15	10

## Data Availability

The original data are available from the corresponding author.
